# Diagnostic Criteria for Parkinson’s Disease: From James Parkinson to the Concept of Prodromal Disease

**DOI:** 10.3389/fneur.2018.00156

**Published:** 2018-03-23

**Authors:** Luca Marsili, Giovanni Rizzo, Carlo Colosimo

**Affiliations:** ^1^Department of Neurology and Psychiatry, Sapienza University of Rome, Rome, Italy; ^2^Gardner Family Center for Parkinson’s Disease and Movement Disorders, Department of Neurology, University of Cincinnati, Cincinnati, OH, United States; ^3^IRCCS Institute of Neurological Sciences of Bologna, Bellaria Hospital, Bologna, Italy; ^4^Department of Biomedical and Neuromotor Sciences, University of Bologna, Bologna, Italy; ^5^Department of Neurology, Santa Maria University Hospital, Terni, Italy

**Keywords:** James Parkinson, Parkinson’s disease diagnostic criteria, Parkinson’s disease guidelines, preclinical Parkinson’s disease, prodromal Parkinson’s disease

## Abstract

The diagnosis of Parkinson’s disease (PD) is based on clinical features and differently to the common opinion that detecting this condition is easy, seminal clinicopathological studies have shown that up one-fourth of patients diagnosed as PD during life has an alternative diagnosis at postmortem. The misdiagnosis is even higher when only the initial diagnosis is considered, since the diagnostic accuracy improves by time, during follow-up visits. Given that the confirmation of the diagnosis of PD can be only obtained through neuropathology, to improve and facilitate the diagnostic–therapeutic workup in PD, a number of criteria and guidelines have been introduced in the last three decades. In the present paper, we will critically re-appraise the main diagnostic criteria proposed for PD, with particular attention to the recently published criteria by the International Parkinson and Movement Disorder Society (MDS) task force, underlying their novelty and focusing on the diagnostic issues still open. We also emphasize that the MDS-PD criteria encompass the two main previous sets of diagnostic criteria (United Kingdom PD Society Brain Bank and Gelb’s criteria), introducing at the same time new aspects as the use of non-motor symptoms as additional diagnostic features, and the adoption of the concept of prodromal PD, crucial to enroll in clinical trials PD patients in the very early phase of the disease. To better understand the real diffusion of the new MDS-PD diagnostic criteria among neurologists, we have also collected selective opinions of sixteen movement disorder experts from various world regions on their practical approach for the clinical diagnosis of PD. Results from this brief survey showed that, although innovative and complete, the revised diagnostic criteria produced by MDS task force are still scarcely employed among clinicians. We believe that both national and international scientific societies should operate in the future for a broader diffusion of these criteria with specific initiatives, including dedicated events and teaching courses.

## Introduction

In the early 19th century, James Parkinson published his essay on “The Shaking Palsy,” in which he described in detail the clinical features (tremor, flexed posture, and festination) of a new disease with an insidious onset and a progressive disabling course called *paralysis agitans*; he was inspired by the observation of various people he noted around the streets of London, all sharing these distinctive physical features (1, 2). A few decades later, Jean-Martin Charcot in Paris first proposed the eponym of Parkinson’s disease (PD) for this disorder, adding extensive details to Parkinson’s observations and identifying bradykinesia and rigidity as key features of the disease, while considering tremor as a typical, but not a mandatory diagnostic feature (2). The diagnosis of PD remains still today a clinical one, and differently from the held belief that detecting PD might be straightforward (3), clinicopathological studies have shown that up one-fourth of patients diagnosed as idiopathic PD during life have a “parkinsonian syndrome” due to an alternative cause (i.e., atypical parkinsonism, Alzheimer-type pathology, or vascular changes) (4). The misdiagnosis rate is higher when only the initial diagnosis is considered (5–7). Conversely, diagnostic accuracy improves during follow-up, with a continuous diagnostic re-evaluation process, when the positive predictive value (PPV) of the last clinical diagnosis prior to death, compared to the autopsy may approach 100% (8).

Given that the confirmation of the diagnosis of PD can only be obtained through neuropathology, to improve and facilitate the diagnostic–therapeutic workup in PD, a number of diagnostic criteria or guidelines (in the US the term practice parameters is also used) have been introduced in the last three decades. Diagnostic criteria provide guidance to clinicians on the specific signs, symptoms, or test results that indicate the presence of an illness, in order to classify patients into diagnostic categories facilitating communication among health professionals. Indeed until the late 1980s, no formal diagnostic criteria for PD were available; at that time different retrospective autoptic studies demonstrated that using only clinical diagnosis without applying formal criteria, the diagnostic accuracy was as low as ~75% (9). During the following years, the application of the diagnostic criteria proposed for PD improved diagnostic accuracy up to 82% (10–13), leaving, however, many unsolved diagnostic challenges. At the same time, different efforts have been employed to draw clinical diagnostic batteries for probable PD and its differential diagnoses, by employing several combined tests including motor function, olfaction, and mood evaluation (14, 15). However, although well designed (with high sensitivity and specificity in discriminating healthy subjects from PD), these diagnostic tests had some intrinsic limitations, due to the absence of ante-mortem “gold standard” other than the opinion of “movement disorder specialists,” to establish the correct diagnosis (14, 15).

The concept of producing guidelines has eventually become popular. Guidelines should provide comprehensive recommendations for the evaluation, diagnosis, treatment, and follow-up of patients with a given disorder and are usually developed by a task force of experts convened by an established scientific society who perform a systematic review of all published evidence on the topic. In this view, the collaborative European Federation of Neurological Societies/Movement Disorders Society-European Section (EFNS/MDS-ES) task force produced initially therapeutic (16, 17) and then diagnostic (18) extensive guidelines for PD among others. The members of this task force reviewed the most relevant publications regarding clinical and laboratory identification of PD, giving specific recommendations to improve the clinical diagnosis (18) still mainly based on United Kingdom Parkinson’s Disease Society Brain Bank clinical diagnostic criteria (UKPDSBB) (10).

Conversely, practice parameters are particular kind of succinct guidelines designed to assist clinicians in providing high-quality assessment and treatment that is consistent with the best available scientific evidence and clinical consensus. The parameters describe generally accepted practices but are not intended to clearly define a standard of care; according to practice parameters, the ultimate judgment regarding the care of a patient is up to the clinician in light of all the clinical evidence on the diagnostic and treatment options. Those published on the diagnosis of PD by the American Academy of Neurology (AAN) (19) represent a good example of how practice parameters address gaps between clinical practice and existing evidence on a given topic (20). Neurologists, patients, and caregivers should keep in mind that the recommendations of the AAN practice parameters are made on the basis of the available pieces of evidence and when good-quality proofs are scarce, direct conclusions are not easily drawn (20). Regarding diffusion among different countries, practice parameters are mainly used in the US, perhaps with the recondite aim to reduce the clinician’s legal responsibilities.

In the present paper, we will critically re-appraise the different diagnostic criteria proposed for PD with particular attention to the recently published International Parkinson and Movement Disorder Society (MDS) diagnostic criteria (21), underlying their novelty and focusing on the diagnostic problems still open. In, addition, we performed an international audit by asking to sixteen movement disorder experts from all over the world, their practical approach toward the clinical diagnosis of PD and their attitude regarding the application of the new criteria.

## Diagnostic Accuracy of Different Sets of Criteria

### From the UKPDSBB to the Gelb Diagnostic Criteria

The United Kingdom Parkinson’s Disease Society Brain Bank (UKPDSBB) represented the first formal diagnostic criteria proposed for PD (10). They consist of a three-step process: step 1 referring to the diagnosis of a parkinsonian syndrome, step 2 referring to exclusion criteria for PD, and step 3 referring to the prospective supportive criteria for PD (10). In step 1, to allow the clinical diagnosis of a parkinsonian syndrome, bradykinesia plus at least one other sign among muscular rigidity, (4–6 Hz) rest tremor, and postural instability are required. In addition (step 2), all secondary causes of a parkinsonian syndrome including history of repeated strokes or head injury with stepwise progression of parkinsonian features, history of definite encephalitis, oculogyric crises, neuroleptic treatment at onset of symptoms, familial history, unilateral features after 3 years, supranuclear gaze palsy, cerebellar signs, early severe autonomic involvement or dementia, unexplained Babinski sign, presence of a secondary cause on imaging, negative response to levodopa and exposure to toxic agents should be excluded (10). Finally (step 3), three or more supportive features among unilateral onset, rest tremor, progression of the disorder, persistent asymmetry, excellent response to levodopa (70–100%), severe levodopa-induced chorea, levodopa response for 5 years or more and clinical course of at least 10 years, should be present (10). According to the UKPDSBB criteria, bradykinesia is the core feature of PD; the clinical spectrum of bradykinesia is quite complex involving not only true bradykinesia (literally “slowness of movement”), but also hypokinesia (literally “less or paucity of movements,” usually employed to indicate decreased amplitude of movements), and akinesia (general lack of movement, including associated and automatic movements). To date, there is a general consensus to use the term bradykinesia to indicate the slowness of initiation of voluntary movement associated with progressive reduction in the speed of repetitive actions (22): using this operational definition, bradykinesia seen in parkinsonism is well differentiated by the pseudo-bradykinesia observed in mimics of parkinsonism (i.e., hypothyroid slowness, dystonia, holding tics, catatonia, and stiff person syndrome).

More than 10 years later, Gelb and other American co-authors (13) published a new set of diagnostic criteria. According to their proposal, the diagnosis of PD requires the presence of at least two cardinal features among rest tremor, bradykinesia, rigidity or unilateral onset. In addition, other causes indicated by the presence of early (within 3 years) postural instability, freezing phenomena or hallucinations unrelated to medications, dementia preceding motor symptoms in the first year, supranuclear gaze palsy, severe dysautonomia, documentation of a condition known to produce parkinsonism (focal brain lesions or neuroleptic use within the previous 6 months), should be excluded (13); the criterion of a considerable and sustained levodopa response is also required (13). Unlike UKPDSBB criteria, Gelb criteria were based on different levels of diagnostic confidence, an approach previously proposed in two sets of criteria, which gained very little diffusion, i.e., Calne et al. criteria (11) and Larsen et al. criteria (12), both suggesting three categories: clinically possible, clinically probable, and clinically definite PD. Gelb and colleagues identified only two clinical diagnostic levels of certainty, a possible and a probable one based on a temporal criterion of symptoms onset; they left the definite diagnosis only when histopathologic confirmation of PD was obtained at autopsy. The most important criticism regarding Gelb criteria is that bradykinesia is not considered an essential feature for diagnosis of PD, whereas this criterion is now considered the most important one in identifying parkinsonism by most of the international experts.

Only one study directly compared sensitivity, specificity, PPV, negative predictive value (NPV), and accuracy of UKPDSBB and Gelb criteria with a definite diagnosis of PD (i.e., using postmortem studies) (23). Diagnostic PPV and NPV were similar in UKPDSBB (92% for PPV and 25% for NPV) and Gelb criteria for possible and probable PD (93% for PPV and 14% for NPV), while specificity was low in both criteria (30–40%). Sensitivity was higher in UKPDSBB (90%) and Gelb criteria for possible PD (87%) compared to Gelb criteria for probable PD (72%). Global accuracy was 84% for UKPDSBB criteria, 82% for Gelb criteria for possible PD, and 69% for Gelb criteria for probable PD (9, 23), with a high number of false positive and false negative cases. In addition, two detailed systematic reviews on the diagnosis of PD carried out in Scotland (24), and Italy (25) were unable to find comparative studies between the two diagnostic criteria and diagnoses of expert clinicians, except for the above-mentioned study by Hughes et al. (23) in which it was only possible to compare PPV (90% for clinical diagnosis versus 92–93% for the two sets of diagnostic criteria). However, a more recent meta-analysis (9) indicated that the accuracy of the clinical diagnosis by an expert is similar (83.9%) to UKPDSBB criteria (82.7%), with a lower sensitivity (81.3 versus 90.8%), but higher specificity (83.5 versus 34%).

Concerning the diffusion of the different criteria, the UKPDSBB criteria have been widely employed in both clinical trials and routine clinical practice around the world, whereas, to the best of our knowledge, only a small number of clinical studies (mainly carried out in the US and in Scandinavia) have adopted Gelb criteria (26–33), somehow justifying their more limited diffusion among practicing clinicians. Calne criteria and Larsen criteria have been used even less.

During the routine use of these two sets of criteria (UKPDSBB and Gelb criteria) spanning for more than two decades, most researchers and clinicians have found several significant limitations (3, 4, 34). First, both criteria focus only on motor features whereas it is now widely accepted that PD is associated with numerous non-motor features more or less responsive to levodopa, including sleep disturbances, mood disorders, autonomic failure, sensory problems, and cognitive impairment. Above all, cognitive impairment is fairly common in PD but, according to the UKPDSBB and Gelb criteria, it might challenge a clinical diagnosis of PD if severe enough to configure dementia within the first year of motor symptom onset. In this case, cognitive impairment becomes an exclusion criterion for PD leading to an alternative diagnosis of dementia with Lewy bodies (DLB) (35, 36). However, the reciprocally exclusive relationship between DLB and PD-dementia remains very controversial, since both disorders present with parkinsonism and dementia, and both are Lewy body disorders and synucleinopathies, leading several experts to think that they should be considered on a spectrum of the same disorder (37, 38). Another noticeable pitfall of UKPDSBB is the idea that genetic risk factors (more than one relative affected by PD) challenge the diagnosis of PD (39), concept not anymore acceptable nowadays.

### Rationale and Construct of the MDS-PD Criteria

In this view, in 2015 the *ad hoc* MDS task force proposed new clinical diagnostic criteria for PD (called the MDS-PD criteria) (21). These criteria were specifically designed for use in research, but they also might be adopted as a general guide to the clinical diagnosis of PD in a routine setting (21). Examination of all cardinal manifestations should be carried out as described in the MDS-Unified Parkinson Disease Rating Scale (UPDRS) (40). In the MDS-PD criteria, the classical signs of the motor syndrome remain the core features of the disease. The essential criterion is the presence of parkinsonism, which is defined as bradykinesia, in combination with at least one between rest tremor (4–6 Hz) and rigidity (21). However, many non-motor manifestations, often dominating the clinical presentation of the disease, have now been incorporated into the diagnostic criteria (21). Based on the assumption that the pathological process of PD may begin in non-dopaminergic structures of the brain or peripheral nervous system, a new diagnostic category has been configured, prodromal PD (21): prodromal PD is considered to represent a true initial stage of PD (41).

The MDS-PD criteria include a three-step process for PD diagnosis. First, parkinsonism is defined and, if the criteria are not met (step 1), prodromal PD or non-clinical PD could then be considered (in addition to other non-parkinsonian tremulous conditions, such as essential or dystonic tremor). Once parkinsonism is diagnosed, the criteria then define whether this is attributable to PD, i.e., when absolute exclusion criteria (step 2) are absent and red flags are balanced by supportive features (step 3) (Table 1). Two levels of certainty are proposed: the diagnosis of clinically established PD requires the absence of absolute exclusion criteria, at least two supportive criteria, and no red flags. When these conditions are satisfied, the expected result is that the large majority of the screened subjects (at least 90%) will have PD, although many true PD cases will not meet this certainty level (21). Differently, the diagnosis of clinically probable PD requires the absence of absolute exclusion criteria, while the presence of red flags is admitted, but this should be counterbalanced by supportive criteria (21). If one red flag is present, there must also be at least one supportive criterion; if two red flags are present, at least two supportive criteria are needed. No more than two red flags are allowed for this category (21). When these conditions are satisfied, the expected result is that at least 80% of patients diagnosed as probable PD truly have PD, but also that 80% of true PD cases are identified (21). Alternatively, if these conditions are not satisfied, clinical PD should not be diagnosed.

**Table 1:** Supportive criteria, absolute exclusion criteria, and red flags for the diagnosis of Parkinson’s disease, according to the revised International Parkinson and Movement Disorder Society (MDS-PD) diagnostic criteria [Postuma et al. (21)].

Supportive criteria
Clear and dramatic beneficial response to dopaminergic therapy. During initial treatment, patient returned to normal or near-normal level of function. In the absence of clear documentation of initial response a dramatic response can be classified as: (a) marked improvement with dose increases or marked worsening with dose decreases. Mild changes do not qualify. Document this either objectively (>30% in UPDRS III with change in treatment), or subjectively (clearly documented history of marked changes from a reliable patient or caregiver) (b) unequivocal and marked on/off fluctuations, which must have at some point included predictable end-of-dose wearing off Presence of levodopa-induced dyskinesia Rest tremor of a limb, documented on clinical examination (in past, or on current examination) The presence of either olfactory loss or cardiac sympathetic denervation on MIBG scintigraphy

**Absolute exclusion criteria: the presence of any of these features rules out PD**

Unequivocal cerebellar abnormalities, such as cerebellar gait, limb ataxia, or cerebellar oculomotor abnormalities (e.g., sustained gaze evoked nystagmus, macro square wave jerks, hypermetric saccades)
Downward vertical supranuclear gaze palsy or selective slowing of downward vertical saccades
Diagnosis of probable behavioral variant frontotemporal dementia or primary progressive aphasia, defined according to consensus criteria within the first 5 years of disease
Parkinsonian features restricted to the lower limbs for more than 3 years
Treatment with a dopamine receptor blocker or a dopamine-depleting agent in a dose/time-course consistent with drug-induced parkinsonism
Absence of observable response to high-dose levodopa despite at least moderate severity of disease
Unequivocal cortical sensory loss (graphesthesia, stereognosis with intact primary sensory modalities), clear limb ideomotor apraxia, or progressive aphasia
Normal functional neuroimaging of the presynaptic dopaminergic system
Documentation of an alternative condition known to produce parkinsonism and plausibly connected to the patient’s symptoms, or, the expert evaluating physician, based on the full diagnostic assessment feels that an alternative syndrome is more likely than PD
**Red flags**
Rapid progression of gait impairment requiring regular use of wheelchair within 5 years of onset
A complete absence of progression of motor symptoms or signs over 5 or more years unless stability is related to treatment
Early bulbar dysfunction: severe dysphonia or dysarthria (speech unintelligible most of the time) or severe dysphagia (requiring soft food, NG tube, or gastrostomy) within first 5 years
Inspiratory respiratory dysfunction: either diurnal or nocturnal inspiratory stridor or frequent inspiratory sighs
Severe autonomic failure in the first 5 years of disease. This can include: (a) orthostatic hypotension—orthostatic decrease of blood pressure within 3 min of standing by at least 30 mmHg systolic or 15 mmHg diastolic, in the absence of dehydration, medication, or other diseases that could plausibly explain autonomic dysfunction, or (b) severe urinary retention or urinary incontinence in the first 5 years of disease (excluding long-standing or small amount stress incontinence in women), that is not simply functional incontinence. In men, urinary retention must not be attributable to prostate disease, and must be associated with erectile dysfunction

**Red flags**

Recurrent (>1/year) falls because of impaired balance within 3 years of onset
Disproportionate anterocollis (dystonic) or contractures of hand or feet within the first 10 years
Absence of any of the common non-motor features of disease despite 5 years disease duration. These include sleep dysfunction (sleep-maintenance insomnia, excessive daytime somnolence, symptoms of REM sleep behavior disorder), autonomic dysfunction (constipation, daytime urinary urgency, symptomatic orthostasis), hyposmia, or psychiatric dysfunction (depression, anxiety, or hallucinations)
Otherwise-unexplained pyramidal tract signs, defined as pyramidal weakness or clear pathologic hyperreflexia (excluding mild reflex asymmetry and isolated extensor plantar response)
Bilateral symmetric parkinsonism. The patient or caregiver reports bilateral symptom onset with no side predominance, and no side predominance is observed on objective examination

*Licensed by Wiley (License number 4167160336642)*.

Given that PD diagnosis is generally clinically based, the MDS-PD criteria are designed to be broadly applicable without the need for ancillary diagnostic testing, although a supportive diagnostic role for these tests is for the first time allowed. Ancillary tests that were included in the criteria include those aimed to document olfactory loss (in the anosmic or clearly hyposmic range, adjusted for age and sex), and cardiac sympathetic denervation as shown by abnormal Metaiodobenzylguanidine single-photon emission computed tomography (SPECT) (21). To be included in the new diagnostic panel, the laboratory marker must have been demonstrated to provide more than 80% specificity in differentiating PD from other parkinsonian conditions, with a minimum of three positive studies from different centers (21). Conversely, among absolute exclusion criteria (Table 1) it is included a normal functional neuroimaging of the presynaptic dopaminergic system. This criterion does not imply that dopaminergic functional imaging is required for diagnosis (nor does the task force wish to imply that this should be routinely performed in diagnosing PD), and if no imaging has been performed, this criterion does not apply (21). To this regard, the new criteria do not leave any more room for the overused term “SWEDD” (i.e., scans without evidence for dopaminergic deficit) to be considered as PD. The currently leading concept is that while an abnormal dopaminergic imaging might suggest clinical or even prodromal PD (for further details, see the paragraph on prodromal PD), a normal dopaminergic imaging unlikely reflects a single clinical entity (42).

As a further consideration, the MDS-PD task force left a loophole to the expert evaluating physician: they stated that PD can still be excluded “… when the documentation of an alternative condition known to produce parkinsonism and plausibly connected to the patient’s symptoms is more likely than PD.” This criterion includes not only rare conditions that can mimic PD, but also the more common degenerative parkinsonian syndromes mimicking PD [multiple system atrophy (MSA), progressive supranuclear palsy, corticobasal degeneration]. Of note is that DLB is not considered an alternative parkinsonian syndrome according to this criterion, given that the MDS-PD criteria do not take into account dementia as an exclusion criterion for PD. Hence, according to MDS-PD criteria and fully in line with the new consensus criteria for DLB (36), for those patients with dementia previously diagnosed as DLB, the diagnosis could be labeled as “PD-DLB subtype.” Unsurprisingly, this recommendation is creating a heated debate in the scientific community (38, 43). A final consideration regarding non-motor features is that MDS-PD criteria are more permissive about the presence of autonomic dysfunction in comparison to the UKPDSBB criteria; they consider severe autonomic dysfunction as a red flag for MSA, only when occurring in the first five years of -disease (21).

Table 2 provides a brief synopsis reporting the main features of the different diagnostic criteria and guidelines developed to improve the diagnosis of PD.

**Table 2:** Synopsis of the main diagnostic criteria proposed for Parkinson’s disease, in terms of methodology, size, and relative diffusion.

Reference	Year of publication	Diagnostic features	Size (no. of pages)	Diffusion
James Parkinson^a^ (1)	1817	Clinical	66	+++
Gibb and Lees^b^ (10) (UKPDSBB)	1988	Clinical	44	+++
Calne et al.^a^ (11)	1992	Clinical	3	+
Larsen et al.^a^ (12)	1994	Clinical	10	+
Gelb et al.^a^ (13)	1999	Clinical	7	++
EFNS/MDS-ES^c^ (18)	2013	Clinical and laboratory	19	++
MDS^a^ (21, 41)	2015	Clinical and laboratory	9	+++(?)

*Type of study: ^a^single- or multi-expert opinion; ^b^Single center clinicopathological study; ^c^Systematic review*.

*EFNS/MDS-ES, European Federation of Neurological Societies/Movement Disorders Society-European Section; UKPDSBB, United Kingdom Parkinson’s disease society brain bank clinical diagnostic criteria; MDS, Movement Disorder Society*.

## MDS-PD Criteria: Open Issues

Parkinson’s disease is classically defined as a distinct clinical entity responsive to dopaminergic therapies, and this therapeutic response has been used to confirm the clinical diagnosis. More recently, many aspects changed, with the expansion of clinical phenomenology and improved understanding of genetic and environmental features that might influence the pathophysiology of the disease at molecular and cellular level (44, 45). Our concept of PD probably need to be expanded to include new clinical syndromes, and in fact, the variability within patients defined as having PD may prompt to identify subgroups sharing discrete clinical, genetic, pathological, and neuroimaging features. In this view, the term “synucleinopathies” is a useful disease concept that includes some parkinsonian syndromes not currently defined as idiopathic PD (as DLB and MSA), but excludes some others that may meet clinical PD criteria (as the parkinsonian syndrome associated with parkin mutations), but have different pathophysiology (44, 46). Others have advocated using the term Lewy body disorders (47): the concept of Lewy body disorders would include DLB, Lewy body PD, rapid eye movement sleep behavioral disorder (REM SBD) and pure autonomic failure, conditions all related to Lewy body pathology (44). Although these are clinically separate entities, the common occurrence of Lewy pathology suggests that they could respond to similar therapies. In sum, a hierarchical classification could be based on different layers as protein pathological deposition (i.e., synucleinopathies including PD, DLB, REM SBD, pure autonomic failure, MSA), type of cellular inclusions (i.e., Lewy body disorders including PD, DLB, REM SBD, and pure autonomic failure) and clinicopathological phenotype (i.e., motor-predominant Lewy body disorder including PD) (44).

Given these postulations, a first possible concern on the MDS-PD criteria is that they are still based on the assumption of PD as a single clinicopathological entity. Conversely, several experts have argued in favor of classifying PD in different subtypes (48–51). As an example, Fereshtehnejad and coworkers (52) have very recently proposed a clinical method for subtyping PD cases on an individual basis, with implications for better patient stratification to be used for personalized medicine. In particular, they investigated a large group of *de novo* PD patients diagnosed according to the current criteria and belonging to a longitudinal international multicenter database. In this study, the authors differentiated three distinct groups of PD patients suggesting that these subtypes differ biologically and in their natural history: a first “mild motor-predominant” group with minor motor and non-motor impairment; a second “diffuse malignant” group with a severe motor and non-motor impairment; and the third group with intermediate features. However, this classification has some limitations. First, it might be only applied on a cohort of *de novo* patients, which is not what routinely seen in clinical practice. Second, these subtypes have not been yet validated by other studies and, third, PD subtypes or clusters cannot be used as a final classification system. Fourth, probably most important in terms of precision medicine is the raising concept of moving away from clinically derived to biomarker-driven subtypes of PD (53). In particular, PD might be considered as a group of disorders that, while related by common dopamine-neuron degeneration, exhibit unique genetic, biological, and molecular abnormalities, which may respond differentially to a given therapeutic approach (53). Following this model, only biomarker-defined, homogenous subtypes of PD might respond to therapies proven to affect the biological processes within each subtype (53).

Another potential issue involves a still neglected subtype of PD that could possibly not be further considered as idiopathic PD using the current criteria: the so-called benign tremulous parkinsonism. This has been considered a distinct clinical phenotype characterized by tremor predominance with a no more than mild progression, except for tremor, and often scarce response of tremor symptomatology to dopaminergic therapy (48); this phenotype in terms of natural history and disability is fairly different from classical PD. Data on the prevalence and the neuropathology of benign tremulous parkinsonism are scarce, but suggest that a portion of these patients (24%) may actually have a non-parkinsonian tremor, as essential or dystonic tremor, since they may not show significant nigral pathology at postmortem examination (54). The identification of these patients in clinical trials looking at possible neuroprotective agents for PD may be critical, since the unbalanced distribution of cases with different natural disease history in treatment groups would alter the trial outcomes. At the moment, the issues of correctly classifying difficult tremulous patients for inclusion in clinical trials might be best addressed by using dopaminergic functional neuroimaging.

The same authors of the MDS-PD criteria (21, 37, 41) underline that a priority task for future research should be to develop a clinical subtype classification and to establish models that satisfy the complexity of early non-motor symptoms such as dementia, taking into consideration controversies to this regard. To do so, imaging and biochemical markers would help to improve diagnostic accuracy and would constitute a bridging element between all clinical, genetic, and ultimately pathological aspects (essential for the diagnostic validation). Priority tasks will be the development of techniques able to detect the pathologic brain α-synuclein deposits using novel imaging brain ligands or searching for extracerebral phosphorylated α-synuclein, e.g., by utilizing skin biopsy (37, 55–57).

### The Perilous Concept of Prodromal PD

Another critical issue is the need to better identify early PD patients to be included in novel neuroprotective trials testing disease-modifying agents. The robust pathological evidence provided by Braak et al. (58) of a slowly progressive/spreading neurodegenerative process, starting from olfactory bulb toward the neocortex throughout the brainstem, suggested the concept of pre- and paucisymptomatic stages of PD, in which early dysfunction of olfactory bulb or brainstem could be detected. Accordingly, it has been proposed that early PD could be divided into three stages: preclinical PD in which the neurodegenerative process is started, without evident symptoms or signs of the disease; prodromal PD in which symptoms and signs of this disease are present, but insufficient to define a full clinical picture of PD; and clinical PD in which the diagnosis of PD is achieved, based on the presence of classical motor signs (according to MDS-PD criteria) (2, 49, 59). At this purpose, in addition to the criteria for the diagnosis of clinical PD, the MDS task force developed specific research criteria for prodromal PD (41). Prodromal disease refers to the stage wherein early symptoms or signs of PD neurodegeneration are present, but a clinical diagnosis is not yet possible. These criteria, which have been developed for research purposes only, include a combination of markers (here the term marker refers to any disease indicator, whether a symptom, sign, or biomarker) ranging from mild motor symptoms [i.e., UPDRS—1987 version (60) score ≥ 3 excluding action tremor; or MDS-UPDRS score > 6 excluding postural-action tremor], non-motor symptoms (i.e., REM SBD, olfactory dysfunction, constipation, excessive daytime somnolence, symptomatic hypotension, erectile/urinary dysfunction, depression), and ancillary diagnostic tests (i.e., abnormal tracer uptake of the presynaptic dopaminergic system: SPECT or positron emission tomography) (41). These criteria represent only a first step in the correct description of early stages of PD, and will require constant updating as more information becomes available (41).

An inherent limitation of this proposal is that markers cannot be combined if they are not independent; as a consequence, no new information is added by identifying two or more markers in the same person (14, 15, 41, 61). As a matter of fact, it is very challenging to determine whether markers are truly independent and this issue has created an animated discussion in the scientific community. A study by Chen and colleagues in 2014 first suggested that the presence of multiple non-motor symptoms in the same person is uncommon in the general population (62). It is indeed difficult to think of a highly prevalent underlying pathology other than prodromal PD in which a person would have combined two or more markers (41). A large longitudinal investigation (HPRO-PD), looking at prodromal PD among participants in the Health Professionals Follow-up Study, has investigated the association of markers as probable REM SBD, constipation, and hyposmia with PD. While constipation, REM SBD and hyposmia are common when considered individually, as expected, their co-occurrence was found to be rare in individuals without PD (63). Again, Fereshtehnejad et al. (64) have investigated in a REM SBD cohort the independence of markers to predict conversion to PD or DLB suggesting that the prodromal criteria could assess with high sensitivity and specificity conversion time and incidence of PD/DLB. More recently, Pilotto et al. (65), when exploring the reliability of MDS research criteria for prodromal PD in diverse prospective cohorts, observed that most patients would not meet the criteria before diagnosis unless testing is performed with specific objective markers (as dopamine imaging and polysomnography to confirm or exclude the diagnosis of probable prodromal PD). Fully in line with these results, researchers from the US PARS study (66) have investigated whether the combination of smell identification testing followed by dopamine transporter (DAT) imaging can identify individuals from the general population at risk for conversion to a clinical diagnosis of PD, concluding that the combination of hyposmia and DAT deficit was highly predictive of conversion to PD within 4 years of clinical follow-up.

Based on these data, prodromal PD criteria represent a promising tool for research purposes, to better investigate the possible risk to develop PD in presymptomatic patients. However, future methods to detect prodromal PD likely will require a multivariate hierarchical approach, and its power will depend on accessing markers over multiple possibly independent domains, such as genetic features, motor and non-motor symptoms, and ancillary diagnostic tests (61). Nonetheless, further investigations are requested to better understand the effective role of the different markers in research studies, such as those which have been recently conducted in REM SBD patients in order to identify new potential α-synuclein biomarkers (56, 57).

## Brief Survey among Movement Disorder Experts

**Figure 1:**
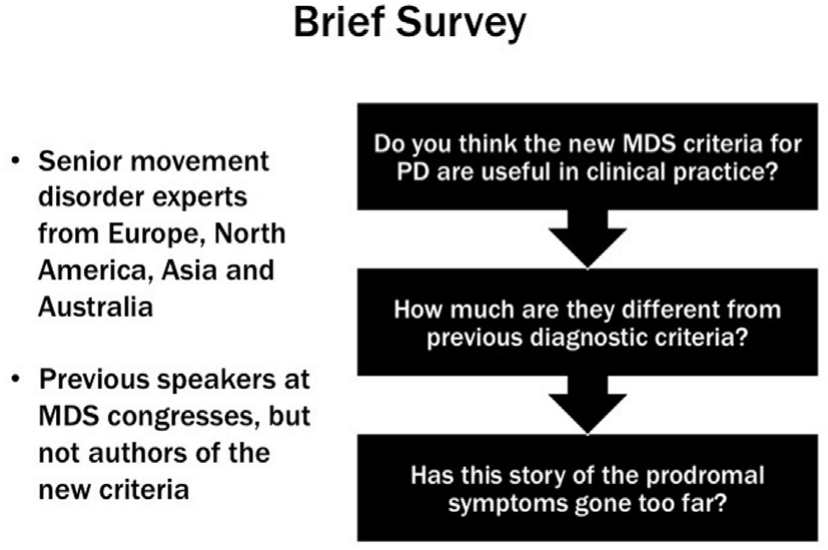
Schematic representation of the three questions posed to the movement disorder experts during the brief survey.

**Figure 2:**
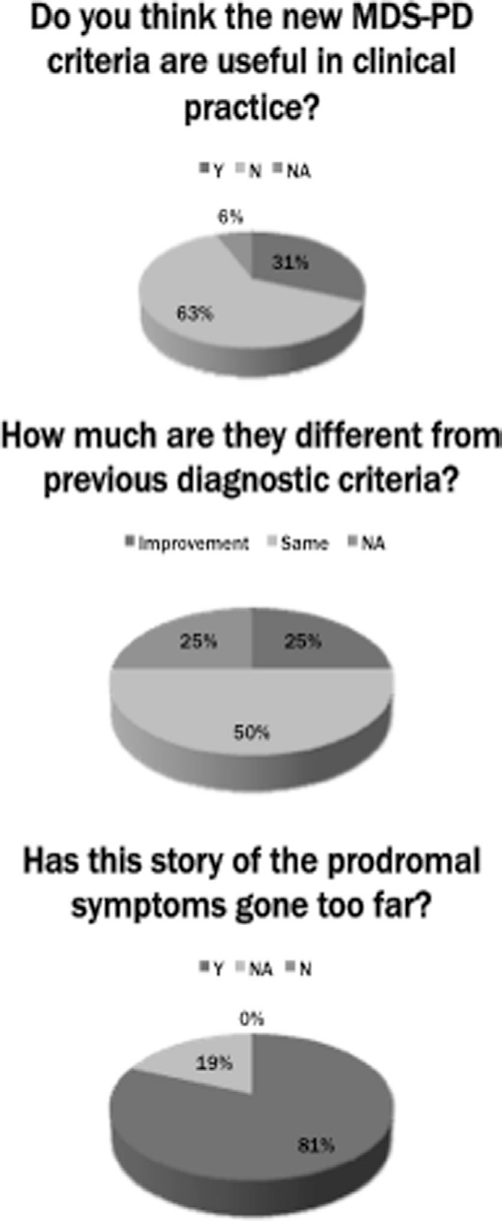
Schematic representation of the senior movement disorder experts’ answers to the three questions of the brief survey. Legend: Y, yes; N, no; NA, not available.

To better understand the *real world* diffusion of the new MDS-PD diagnostic criteria, we conducted an international survey by asking to sixteen senior movement disorders experts (all regular speakers at international congresses, but not authors of the new criteria) their practical approach to the clinical diagnosis of PD, and whether they have applied the new criteria in their daily practice. The experts were based in Europe (*n* = 9), North America (*n* = 2), Asia (*n* = 2), and Australia (*n* = 3). The survey was conducted by email and consisted of two straightforward questions: (1) “Do you think the new MDS-PD criteria are useful in clinical practice?” (2) “How much do you think are they different from previous diagnostic criteria?” And a third “provocative” question: (3) “Has in your opinion this story of the prodromal symptoms gone too far?” (Figure 1). Interestingly, among the sixteen experts consulted, only five answered affirmatively to the first question, one colleague did not answer because he did not know in details the new criteria, and the other ten considered the new MDS-PD criteria not so valuable in the clinical setting. Again, when answering to the second question, eight colleagues considered these criteria quite similar to the previous UKPDSBB criteria, two colleagues considered these criteria too complex and artificial, two colleagues did not answer directly at this question and only the remaining four considered the new criteria to represent a real improvement, particularly for the attention given to the role of non-motor features of PD. When asked to the last challenging question, three experts did not answer directly and the other thirteen were fundamentally skeptical. In particular, although it is well known that the prodromal criteria have been clearly developed for research purpose only, they argued that a possible misuse of these criteria could lead to the amplification of the role of prodromal symptoms also in a routine clinical setting, stating that the best approach is to clinically monitor subjects at risk for PD with annual or biannual visits. In addition, when asked to add some other comments or observations about the new MDS criteria, three colleagues expressed a distinctly positive opinion for the introduction of “red flags” and of supportive criteria (“green flags”), underscoring the possible benefit for research and future neuroprotective trials. A few of them expressed their disappointment toward the inclusion of “normal functional neuroimaging of the presynaptic dopaminergic system” as an absolute exclusion criterion (given that low-quality dopaminergic imaging might result negative also in early PD patients); others felt that the MDS criteria suffer from excessive sketchiness (referring to the concept that “the number of red flags should be equal to, or less than, the number of supportive criteria” for probable PD). In sum, they argued that the basic criteria for diagnosis (i.e., levodopa response with no “red flags”) should still remain the crucial part of the “core” diagnostic criteria initially, no matter how clinicians wish to modify the other criteria or add on more clarifications to the criteria (Figure 2).

This informal brief survey provides current simple information about “real-life” differences in the approach to the clinical diagnosis of PD among clinicians. Possible limitations of the here reported international audit consist the restricted number of participants and that only experts not directly involved in the production of the criteria have been interviewed, configuring a possible negative bias. In summary, we suggest that the diffusion of the new criteria should be better implemented not only among general neurologists, but also among movement disorders specialists. Although the revised MDS-PD criteria have been published since 2015, many colleagues do not apply these criteria in routine clinical practice because of scarce knowledge and probably also for some prejudices.

## Conclusion

Since the first description of PD two centuries ago, our understanding of this disorder has increased at different levels, from a more accurate definition of its clinical features and pathophysiological mechanisms, to the characterization of its neuropathological hallmarks necessary for the diagnostic confirmation (67). To do so, a great contribution was achieved through the introduction of UKPDSBB criteria, that represent a relatively simple tool to increase the accuracy of diagnosis; the Gelb criteria deserve, however, the merit to have introduced the distinction between a possible and a probable diagnosis of PD. The recent MDS-PD criteria encompass the two previous sets of criteria introducing also new important aspects as the use of non-motor symptoms as possible diagnostic features and the adoption of the concept of prodromal PD, fundamental for research studies (to correctly enroll early PD patients in future trials). We emphasize that, although innovative and complete, the MDS-PD criteria still lack of the pathological validation and are scarcely employed among clinicians, so far. We think that both national and international scientific societies should operate in the future for a broader diffusion of these criteria with specific initiatives, including dedicated events and teaching courses.

## Author Contributions

LM: conception, organization, and execution of the review project; writing, review, and critique of the manuscript. GR: organization and execution of the review project; critique of the manuscript. CC: conception, organization of the review project; review and critique of the manuscript.

## Conflict of Interest Statement

CC received honoraria for speaking/advisory boards from Ipsen, Zambon, Sunovion, and Bial. The remaining authors declare that there are no additional disclosures to report.

## Abbreviations

PD, Parkinson’s disease; EFNS/MDS-ES, European Federation of Neurological Societies/Movement Disorders Society-European Section; UKPDSBB, United Kingdom Parkinson’s disease society brain bank clinical diagnostic criteria; AAN, American academy of neurology; SPECT, single-photon emission computed tomography; SWEDD, scans without evidence for dopaminergic deficit; REM SBD, rapid eye movement sleep behavioral disorder; MSA, multiple system atrophy; DLB, dementia with Lewy bodies; PPV, positive predictive value; NPV, negative predictive value; MDS-PD criteria, movement disorder society clinical diagnostic criteria for Parkinson’s disease; UPDRS, unified Parkinson’s disease rating scale; MDS-UPDRS, movement disorder society-sponsored revision of the unified Parkinson’s disease rating scale; PET, positron emission tomography; DAT, dopamine transporter.
